# Synthesis of Electrical Conductive Silica Nanofiber/Gold Nanoparticle Composite by Laser Pulses and Sputtering Technique

**DOI:** 10.1186/s11671-017-2200-z

**Published:** 2017-06-30

**Authors:** Sarah Hamza, Anna Ignaszak, Amirkianoosh Kiani

**Affiliations:** 1 Department of Mechanical Engineering, Silicon Hall: Laser Micro/Nano Fabrication Facility, University of New Brunswick, New Brunswick, E3B 5A3 Canada; 2 Department of Chemistry, University of New Brunswick, New Brunswick, E3B 5A3 Canada; 3 Department of Automotive, Mechanical and Manufacturing Engineering, University of Ontario Institute of Technology (UOIT), Oshawa, ON Canada

**Keywords:** Nanomaterials, Silicon, Laser materials processing, Biological sensing and sensors, Materials and process characterization, Nanostructure fabrication

## Abstract

Biocompatible-sensing materials hold an important role in biomedical applications where there is a need to translate biological responses into electrical signals. Increasing the biocompatibility of these sensing devices generally causes a reduction in the overall conductivity due to the processing techniques. Silicon is becoming a more feasible and available option for use in these applications due to its semiconductor properties and availability. When processed to be porous, it has shown promising biocompatibility; however, a reduction in its conductivity is caused by its oxidization. To overcome this, gold embedding through sputtering techniques are proposed in this research as a means of controlling and further imparting electrical properties to laser induced silicon oxide nanofibers. Single crystalline silicon wafers were laser processed using an Nd:YAG pulsed nanosecond laser system at different laser parameters before undergoing gold sputtering. Controlling the scanning parameters (e.g., smaller line spacings) was found to induce the formation of nanofibrous structures, whose diameters grew with increasing overlaps (number of laser beam scanning through the same path). At larger line spacings, nano and microparticle formation was observed. Overlap (OL) increases led to higher light absorbance’s by the wafers. The gold sputtered samples resulted in greater conductivities at higher gold concentrations, especially in samples with smaller fiber sizes. Overall, these findings show promising results for the future of silicon as a semiconductor and a biocompatible material for its use and development in the improvement of sensing applications.

## Background

Biocompatible sensing materials tend to be costly to produce as well as having a low signal-to-noise ratio (SNR); A signal-to-noise ratio is a measure of signal power to a level of noise power (background noise) and is expressed as a measurement of decibels (dB). Nanomaterials were introduced as an attempt to reduce the muffling caused by the noise. Two main methods are used to reduce muffling, namely, carbon nanotube formation and nanomaterials [1]. The success of carbon nanotubes as sensors may be attributed to their increased effective surface area, which decreases the electrode impedance and increases current [1–4]. The increased surface area also immobilizes more enzymes thereon in biomedical applications [2]. However, there are some disadvantages to fabricating carbon nanotubes. For instance, it is expensive and has low purity, a shortage in alignment control, a lack of aqueous solubility, and a high reactivity caused by dangling nanotubes [5].

Adverse tissue reactions and resistance to degradation are important biocompatibility factors [6]. Porous silicon, which is formed of a unique structure of nanocrystallites and pores, exhibits properties that are valuable for its use as a biomaterial and potential biosensing applications [7]. Silicon—a commonly used material—is versatile in contemporary microprocessing techniques because of its availability and low cost [8, 9]. Silicon can be processed to form macro, micro, and nanopores. The ideal pore diameter for biocompatible sensing devices is between 2 and 50 nm. These pore sizes enable biomolecular diffusion and larger surface exposure, resulting in increased biomolecule immobilization compared to 2D surfaces and make it an excellent material for biosensing applications [8].

Various methods can be used to modify the surface of silicon substrates to fabricate silicon-based sensors. Electrochemical etching is used in many cases to modify silicon into a porous structure. This method requires the use of various chemicals and specialized equipment. The procedure initially requires thorough cleansing of the wafer. Certain chemicals may highly react to defects in the structure of silicon and release toxic gases [9, 10]. Electrochemical etching also strongly influences the surface topography, making it more difficult to control [11]. Achieving a uniform porous surface using this technique is complex and highly dependent on and sensitive to the etching parameters, also resulting in the production of large quantities of waste [12]. Moreover, a high concentration of hydrogen bonds subsides on the surface post preparation, making it highly unstable [8]. Photolithography is another method for modifying the surface of silicon substrates in order to fabricate a biocompatible silicon-based sensor [13, 14]. This method enables the patterning and control of cell behavior. Its main disadvantage is that due to the optical diffraction of the light beam, the resolution is limited to a maximum of 1 μ in practice.

Laser processing is another method for modifying the surface of silicon substrates. It is used to optimize a material’s performance such as its absorption, susceptibility to wear, surface chemistry, and crystal structure. Surface properties can be controlled in this manner without affecting the bulk of the material [8, 9].

The addition of gold nanoparticles is an attractive method for modifying the surface of silicon substrates in order to fabricate a silicon sensor. Gold nanoparticles have important properties including their conductivity, high surface-to-volume ratio, excellent molecular recognition, and high surface energy [15, 16]. Their unique chemical and physical properties help to transfer electrons from the biospecific layer to the electrode surface [15]. Gold nanoparticles also increase the sensitivity of biochemical detection of electrochemical biosensors [17, 18].

Previously published results by Colpitts and Kiani have proven the usage of a nanosecond pulsed laser system in the formation of biocompatible fibrous structures on silicon [12, 19]. Their initial results inspired the aim of this research to propose a method of customizing the properties of laser processed silicon to improve its viability in future biological sensing applications which require properties of both biocompatibility and electrical conductivity. Also outlined is an effective method of generating nanofibrous silicon oxide using a commercial nanosecond pulsed laser. This involved the processing of a crystalline silicon wafer using an Nd:YAG nanosecond pulsed laser at a constant power of 12 W with a variation in the overlaps (number of laser beam scanning through the same path) and line spacings (distance between scanning paths). Gold sputtering was then conducted on its surface for duration of either 4 or 8 min. Changes in absorption and conductivity as well as surface topography were investigated and are discussed.

## Materials and Methods

**Fig. 1:**
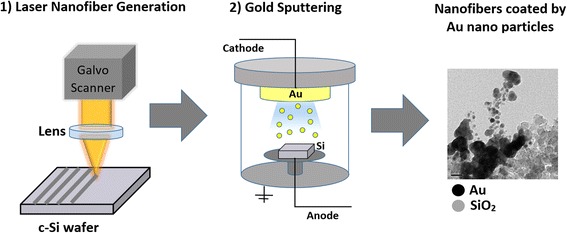
Approach I: gold sputtering of laser generated silicon oxide nanofibers

This approach involved the laser processing of single crystalline silicon wafer <100> at an average power of 12 W, at line spacings of 0.025, 0.1, and 0.15 mm, and at either one, three, or five overlaps. The line spacing refers to the space between each successive line pulsed by the laser, measuring from the center of the laser beam. The overlaps (OL) denote the number of pattern repetitions made on the surface of the silicon, for example, three overlaps would mean that the laser beam passes over the ablated line three times. These were gold sputtered for either 4 or 8 min. Fig. 1 illustrates the overall process.

### Laser Processing

An Nd:YAG nanosecond pulsed laser with a wavelength of 1064 nm was used for this experiment. The circular output beam of the laser has a diameter of 9 mm and is reduced to 8 mm using an iris diaphragm before entering an XY galvanometer scanner (JD2204 by Sino-Galvo). This scanner has an aperture of 10 mm and a beam displacement of 13.4 mm. A F-theta lens with a focal length of 63.5 mm was used to control the focus of the laser on the sample surface, resulting in a theoretical laser spot diameter of 20 μm. EZCAD software was used to control laser parameters, e.g., to specify scanning speeds, overlaps, frequency, and line patterning.

### Microscopy and Surface Characterization: Scanning Electron Microscope (SEM) and Scanning Transmission Electron Microscope (TEM) and Energy Dispersive X-Ray (EDS)

A variety of means were employed for surface characterization, including a JEOL JSM-6400 scanning electron microscope (SEM) mounted with an EDAX Genesis 4000 energy dispersive X-ray (EDS) and A JEOL JEM-2010 scanning transmission electron microscope (TEM) adapted with a Gatan UltraScan camera using DigitalMicrograph was used to collect the desired images.

### Light Spectroscopy

The STS-NIR Spectro Radiometer (Ocean Optics, Dunedin, Florida, USA) was used to determine the optical properties of the samples, namely, to measure the reflectivity coefficient of the samples at varying overlaps and line spacing at wavelengths between 175 and 885 nm and an optical resolution of 1.5 nm [19].

### Impedance Spectroscopy

A CH Instruments Inc. (USA) model 760 potentiostat was used to measure the conductivity of the processed silicon samples using AC impedance spectroscopy. The samples were connected via alligator clips to the spectrometer (two-electrode mode) and measurements were obtained at frequencies between 0 and 1 × 10^6^ Hz and at the potential amplitude of 10 mV.

### Image Analysis

The software ImageJ 1.501 by Wayne Rasband at the National Institutes of Health, USA, is used to determine particle and fiber diameters. It allows for the manual import and measurement of the features captured by SEM and TEM images.

## Results and Discussion

### Generation of Nanofibrous Structures

Silicon samples were processed at one, three, and five overlaps at an average power of 12 W with line spacings of 0.025, 0.10, and 0.15 mm. SEM images were collected to determine the type of nanostructures present.

**Fig. 2:**
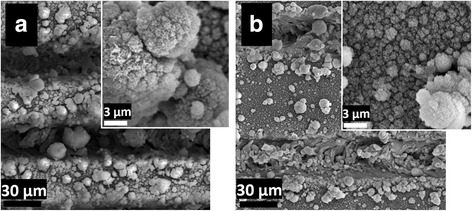
SEM images of laser processed silicon at five overlaps (OL) with line spacing of **a** 0.1 mm, **b** 0.15 mm

Increasing the line spacing resulted in the formation of microparticles with nanoscale porosity rather than nanofibers. The laser-ablated area is distinctly apparent in both (a) and (b) of Fig. 2, as expected since the laser spot diameter is of approximately 0.02 mm and much smaller than the designated line spacing. At 0.1 mm, microparticles form on the surface between the laser-ablated regions. Higher magnification reveals these microparticles to be formed of fine fibrous structures. At 0.15 mm, the microparticles are smaller and sparser, with a higher density of nanoparticles forming on the surface instead. The porosity of the nanostructures differs from the larger microparticles. The microparticles at the 0.15-mm line spacing have a denser structure compared to the 0.1 mm sample. In theory, it is expected that an increase in laser plasma plume temperature will result in particle growth [20], as may be observed by comparing the images in Fig. 2.

**Fig. 3:**
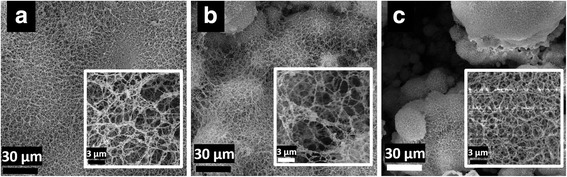
SEM images of laser processed silicon at 0.025 mm line spacing. From *left* to *right*, the overlap changes from one, three, and five, respectively (the npannel within each image shows high magnification SEM image)

The SEM images in Fig. 3 show (a) a uniform dispersion of interlinked nanofibers that form at a line spacing of 0.025 mm. When the overlap number is increased to three, (b) small clusters of nanofibrous particles begin to form. By the fifth overlap, (c) clear clusters of nanofibrous structures form with spaces between them. Again, increasing overlap is expected to increase particle growth because of increased temperature and light absorption. An increase in fiber diameter was also observed with increasing overlaps. Based on SEM images, the fiber diameters were analyzed using an image visualization software ImageJ 1.501 developed by Wayne Rasband at the National Institutes of Health, USA. The smallest fiber diameter—an average of 75 nm—was observed at one overlap. Literature indicates that nanoporous structures increase the biocompatibility of the material by affecting the topology and cell scaffolding [21].

It is not surprising that optimal nanofiber generation was observed at the smallest line spacing of 0.025 mm. Since the laser diameter is theoretically very close to the size of this line spacing, little to no area is left that does not come into direct contact with the laser. This results in a more heated region and the plume density is kept stable for a longer period. This further increases the overall light absorption of the sample due to the change in topography. By creating a fiber network, the surface area increases and therefore all the mechanisms directly linked to area are enhanced.

Removing material from a solid surface using pulsed laser technology can induce nanoparticle formation. When the laser is shined on a surface, it induces vaporization and removes atoms from the bulk surface, thus enabling the laser pulse to go deeper into the material. Laser depth depends on factors such as its wavelength and the material’s physical properties. The laser’s electromagnetic fields eject electrons by discharging energy and momentum on the surface of the material. The energy transfer involved in the laser’s interaction with the material causes its temperature to rise, which in turn causes the formation of an ionized gas known as plasma that will expand like a shockwave around the laser’s focus. Particles are removed from the surface when the laser’s intensity (fluence) is greater than the material’s ablation threshold. The contents of the plasma take the shape of a plume: a region containing a mixture of ions, electrons, and nanoparticles that are highly reactive. When laser ablation is conducted in air, oxidation of the ejected particles may result. As the plume expands, its extremities are cooler than its core [22]. As a result, newly formed particles move toward cooler regions, which causes them to supersaturate, further nucleate, and crystallize into a solid structure. Collisions between gas atoms and the ablated plume in the thin interface layer generate nanoparticles and aggregates. The ambient gas coalesces with the evaporated atoms and ions at high temperatures. As the plume cools, aggregate formation begins. By the end of the laser pulse, aggregate-aggregate and atom-aggregate attachments occur [23].

**Fig. 4:**
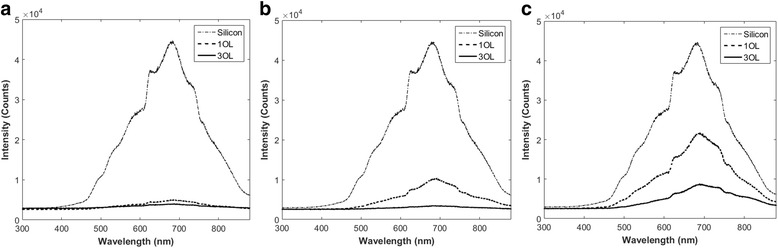
Light reflection of laser-treated silicon samples at one and three overlaps (OL) and at line spacings of **a** 0.025, **b** 0.10, and **c** 0.15 mm

Light absorption coefficients were experimentally determined through light spectroscopy. As Fig. 4 shows, the closer line spacings resulted in much lower reflectivity due to the increased overall surface roughness. As mentioned previously, higher overlap number increases light absorption. From these theoretical values, the maximum was found in each case, and this value was used to determine a precise reflectivity coefficient.

**Fig. 5:**
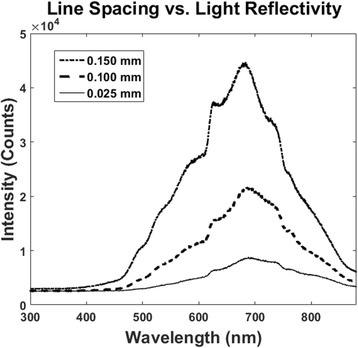
Light reflection of laser-treated silicon samples at one overlap (OL) and line spacings of 0.025, 0.10, and 0.15 mm

The effects of line spacing on reflectivity were also studied (Fig. 5). Comparing the results from one overlap, increase in line spacing resulted in a much higher reflectivity. As expected, porous and fibrous silicon absorbed more light than silicon showing signs of microparticles alone. At larger line spacings, portions of the silicon were not laser ablated; instead, microparticles resting on a smoother surface remained, which exhibited reflective properties more akin to those of unprocessed silicon.

Once the incident light enters the material, the absorption causes a reduction in the light intensity as the depth increases based on the material’s absorption coefficient, *α*. Assuming a uniform material with a constant *α*, the intensity, *I*, decays with depth *z* follows the Beer-Lambert law, where *I*_0_ represents the intensity inside the surface after considering reflection losses [24].

1 $$ I(z)={I}_0{e}^{\hbox{-} \alpha z} $$

Laser ablation is highly dependent on the heat transfer to the material. With nanosecond lasers, it is generally assumed that most of the absorption is due to single photon interactions. Increases in light absorption result in higher temperatures and plume pressures [25], which encourage nanofibrous structure formation.

When the thermalization rate is greater than the laser-induced excitation rate, the process is referred to as photothermal or pyrolytic, where the absorbed laser energy is assumed directly transformed into heat. This is the case when the laser pulse times are greater than the nanosecond range. Photothermal processing leads us to the modeling of heat flow through the material. Its response to the laser is due to thermal effects in both its temporal and spatial coordinates and can be modeled from derivations of the heat equation.

In order to mathematically determine the expected average temperature relationships between the sample surfaces, the maximum temperature, *T*, occurring at the end of the laser pulse (*t*_*p*_), is determined through a one-dimensional model as follows [12, 26, 27]:

2 $$ T\left(0,\ {t}_p\right)=\sqrt{\frac{2 a}{\pi^3{t}_p}\frac{4 K\left(1- R\right) P}{kf{ d}^2}} $$

The laser pulse duration (*t*_*p*_) in our case was of 57.5 ns, with a spot diameter (*d*), 20 μm at a frequency *(f)* of 100 kHz, and an average power (*P*) of 12 W. The thermal diffusion coefficient (*a*)––for silicon was set at 0.000085 m^2^/s the residual energy coefficient *K* was set at a constant of 0.8 for silicon, and the thermal conductivity *k* at 155 W/mK. *R* in this case is the reflectivity values experimentally determined above. From this, the average surface temperature after *n* pulses was calculated according to Eq. 3 below, where *α* is a constant of the square root of the frequency multiplied by the pulse duration ($\alpha =\sqrt{t_p f}$) [12, 26, 27].

3 $$ {\overline{T}}_n=2\alpha \frac{\left[1-\frac{2}{3}\alpha \right]}{\left(1+{\alpha}^2\right)}\frac{T_m}{\left(1-\alpha \right)}\left[1+\frac{\alpha^n-\alpha}{n\left(1-\alpha \right)}\right] $$

**Fig. 6:**
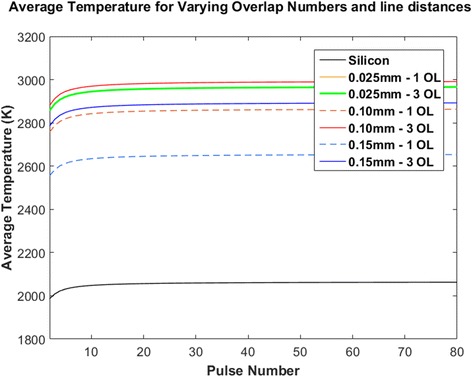
Theoretical average temperatures reached by laser-ablated single crystalline silicon samples after a given number of pulses at a single point

Using the above Eqs. 2 and 3 and assuming no particle evaporation, plots of the average surface temperatures reached by the samples for line spacings of 0.025, 0.10, and 0.15 mm at one and three overlaps were generated as seen in Fig. 6.

From the temperature profiles shown in Fig. 6, there is a gradient that forms before the maximum average temperature is reached. This gradient causes the formation of the plasma mentioned earlier. The steady state maximum values for each of the samples were determined and as concluded, the samples at three overlaps reached a higher average surface temperature than those at one overlap. This can be explained by the increase in the size of the nanoparticles, hence leading to higher absorptions. The only exceptions are the samples at a line spacing of 0.025 mm, where both these samples resulted in the same maximum average temperature. This is due to the very close correlation in their reflectivity values.

The average number of particles evaporated from the surface by successive pulses was theoretically estimated based on laser processing parameters and material properties. The evaporation rate, *R*_evp_, by single pulse ablation is calculated in the one-dimensional model as follows [20, 27]:

4 $$ {\left\langle {R}_{\mathrm{evp}}\right\rangle}_{\mathrm{therm}}={n}_{\mathrm{air}}{\left(\frac{A{ k}_B{a}^{\frac{1}{2}}{t}_p^{\frac{1}{2}}{t}_{\mathrm{eq}}{P}_{\mathrm{avg}}}{M_a k{\pi}^{\frac{3}{2}}{R}_{\mathrm{rep}}{A}_{\mathrm{foc}}}\right)}^{1/2} $$

Here, *n*_air_ is density of air (kg/m^3^), *A* is absorption coefficient, *t*_eq_ is equilibration time, *P*_avg_ is average power, *M*_*a*_ is atomic mass (kg), *A*_foc_ is focal area, *R*_rep_ is frequency, and *k*_B_ is Boltzmann constant (J/K). Using this and converting the rate into a number of atoms based on the atomic mass of silicon, the average number of evaporated particles can be estimated as [20, 27]

5 $$ {N}_{\mathrm{MP}}={R}_{\mathrm{evp}}{R}_{\mathrm{rep}}{A}_{\mathrm{foc}}{D}_t $$

**Fig. 7:**
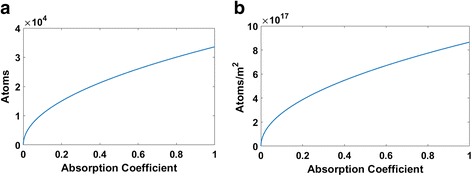
Theoretical number of evaporated atoms by single and successive pulses at varying absorption coefficients. **a** number of Evaporated Atoms by Successive Pulses; **b** number of Evaporated Atoms by Pulse Ablation

The parameters used in this case were as described in the previous equation; however, the equilibration time, *t*_eq_ was set to 1.5 × 10^10^ s, the laser frequency *R*_rep_ at 100 kHz, the pulse dwell time *D*_*t*_ calculated from the effective number of pulses, and finally, the focal area *A*_foc_ was calculated from the theoretical minimum laser spot diameter. Values were determined for both the evaporation rate and the estimated number of evaporated particles at different laser absorption coefficients. The results are graphically shown in Fig. 7.

As absorption increases, the average number of particles as well as the rate begins by increasing seemingly parabolically. There is a rapid increase in the number of evaporated particles at lower absorption values. Although a higher number of atoms can be achieved as the absorption increases, the curve no longer grows as rapidly. This explains why silicon laser processed surfaces with higher absorption coefficients are more likely to have nanoparticles and fiber formations as the number of evaporated atoms increases, thus allowing for more structural rearrangement.

### Gold Sputtering of Laser-Generated Silicon Oxide Nanofibers

The samples prepared at an average power of 12 W and at a line spacing of 0.025 mm were sputtered with gold to assess their conductive properties. Samples were gold sputtered for either 4 or 8 min. The conductivity and particle size effects were measured and compared at different overlaps.

**Fig. 8:**
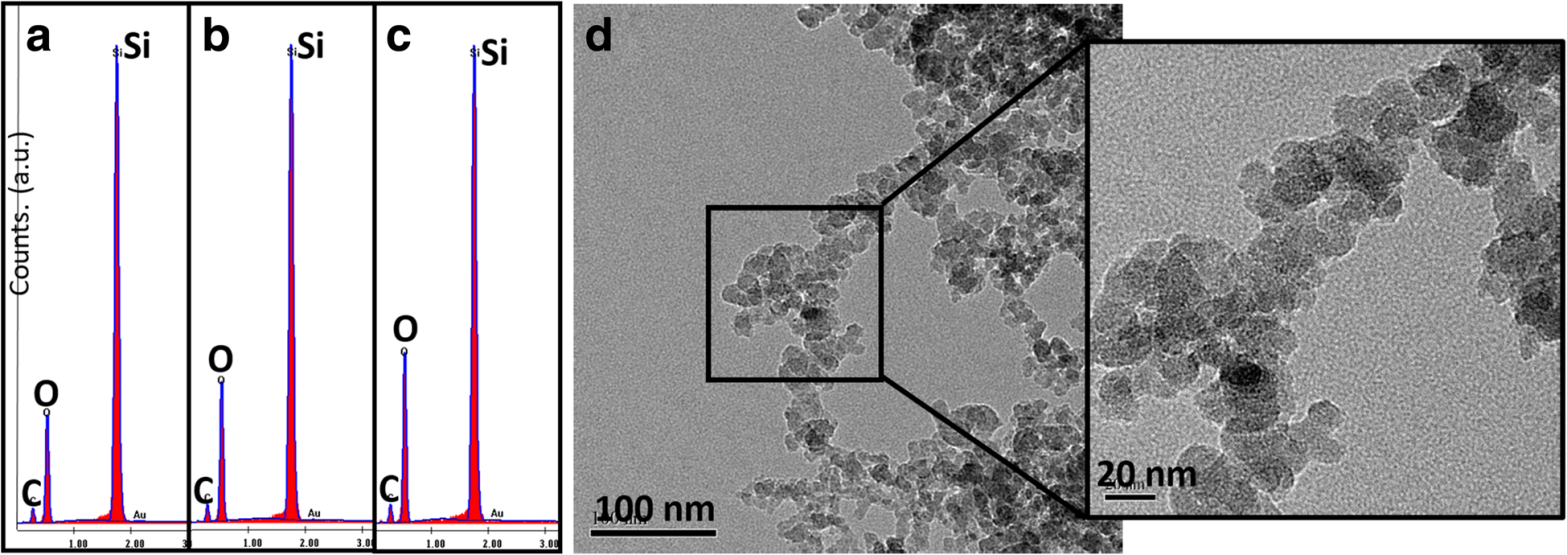
EDX images of laser processed silicon at 0.025 mm. **a** 1 OL, **b** 3 OL, **c** 5 OL, **d** TEM of sample prepared at five overlaps (OL)

In previous studies, it has been proven that the oxidized coating markedly influence biocompatibility by increasing adsorption of hydroxyl groups, lipoproteins, and glycolipids. Fig. 8 shows the EDX and TEM results of the 0.025-mm ablated silicon samples. The oxygen concentrations are seen to rise with increasing overlap numbers (a–c) suggesting increased biocompatibility. The highest count for oxygen is seen in the sample with the highest absorption, namely, at five overlaps (c). As the nanofibrous generation, overall average temperature, and number of evaporated atoms of a sample increase, there are more particles interacting with the ambient air in which the ablation is being conducted. This results in particles rich in oxygen due to the oxidation reactions occurring within the laser plume.

**Fig. 9:**
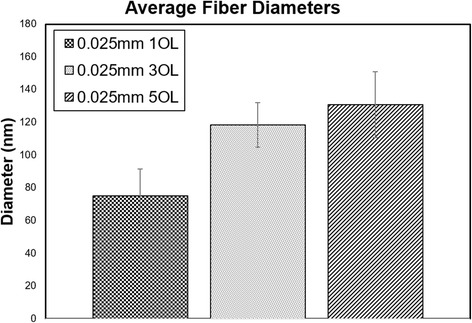
Average fiber diameters calculated from SEM images at one, three, and five overlaps (OL)

SEM images were analyzed using ImageJ to determine the approximate diameters of the fibers occurring at the 0.025-mm line spacing. As referenced in Fig. 9, the fiber diameters grow larger as overlaps are added. From above, we know that the oxygen levels increased with the addition of overlaps, hence partially explaining the growth in size of the fibers.

**Fig. 10:**
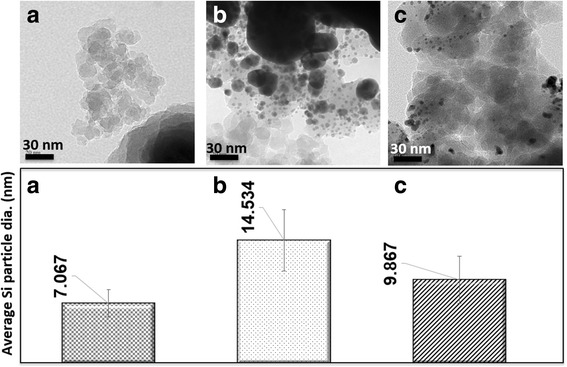
Average silicon particle diameters of samples gold sputtered for 8 min. **a** 1 OL 0.025 mm, **b** 5 OL 0.025 mm, **c** 5 OL 0.15 mm

Through TEM images, the average gold and silicon particle diameters were calculated along with their standard deviations. As the number of overlaps was increased, it was found that the average silicon particle diameter grew as well. This agrees with both the theory that particle growth occurs with increasing absorption and with the previous results showing the expansion of fiber diameters with added overlaps. The fiber diameter increase can be explained by the growing particle sizes of the silicon. As shown in Fig. 10, the sample with five overlaps and a line spacing of 0.025 mm has the largest silicon particles compared to the samples at shorter line spacings. This is also the sample with the highest absorbance value compared to the other two samples. This explains the larger fiber diameters seen in sample b of the figure compared to sample a. The sample shown in c has an absorbance coefficient that falls between those of samples a and b, thus explaining its particle growth compared to that of the other two samples.

**Fig. 11:**
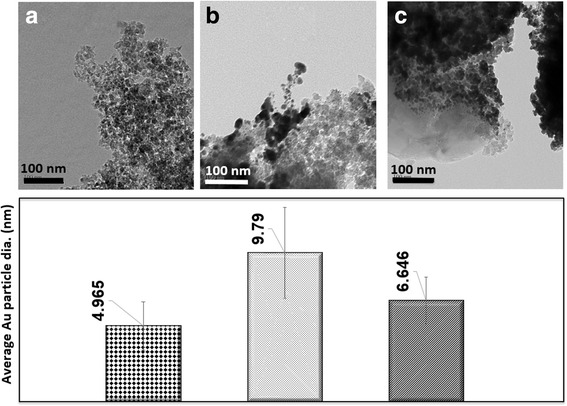
Average gold particle diameters in nanometers of samples gold sputtered for 8 min. **a** 1 OL 0.025 mm, **b** 5 OL 0.025 mm, **c** 5 OL 0.15 mm

In Fig. 11, the diameters of gold particles are shown to have a very similar growth pattern to that of the silicon particles. As the overlap number is increased, the gold particle diameters are seen to increase as well.

**Fig. 12:**
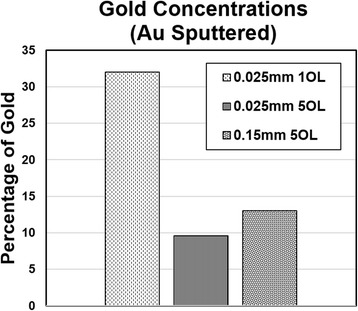
Gold concentrations found on laser processed silicon sputtered with gold for 8 min at 1 OL 0.025 mm, 5 OL 0.025 mm, and 5 OL 0.15 mm

The gold concentration in each sample was estimated using the ImageJ software (Fig. 12). The concentrations were found to decrease at 0.025 mm when going from one to five overlaps. From the SEM images and fiber diameters, the silicon sample at one overlap and 0.025-mm line spacing has thinner fibers and hence less closely packed spaces. This would allow for more gold particles to fall between these spaces and attach separately around the fibers as opposed to agglomerating. At five overlaps, the fibers are much thicker and the spacing between them is smaller, allowing for fewer gold particles to deposit into the crevices. At a 0.15-mm line spacing and five overlaps, the concentration fell between that of the two previously discussed samples as shown in Fig. 10. When comparing the line spacings, an increase in the latter results in a decrease in gold concentrations. The reduction causes the absorbance of the material to decrease and hence reduces particle growth. At five overlaps, the gold concentration increases when the spacing increases because the surface is smoother (smaller overall contact area) which results in higher gold concentration in synthesized nanofibrous structures.

Theoretically, longer pulse durations and higher plume density and temperatures result in larger nanostructure formation. Nanostructure sizes depend highly on the plume diffusion time scale while their type depends on the density of the evaporated atoms. For this reason, to achieve nanofibrous structures, the laser pulses must be kept continuous for the plume density to remain at the critical level required for their formation. Hence, the larger particle sizes with growing overlaps can be explained in this fashion due to the higher overall surface temperatures and absorption coefficients [24].

**Fig. 13:**
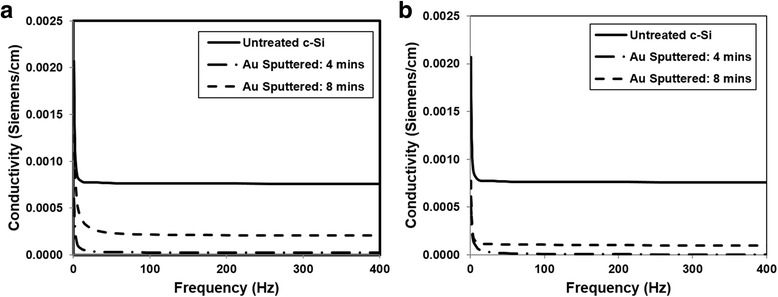
The total conductivity of gold sputtered silicon samples. **a** 1 OL, **b** 2 OL (higher conductivity of untreated silicon is due to its zero porosity)

The overall conductivity was measured through impedance spectroscopy for samples with one and two overlaps at a line spacing of 0.025 mm. The conductivity was measured using larger square samples of approximately 1.5 × 1.5 cm and connected directly to the spectrometer (in order to minimize the contact resistance). The Bode diagrams (an absolute total resistance as the function of AC frequency) were used to calculate the specific conductivity of films (in Siemens per centimeter, S/cm) after standardization to their thickness and area. Fig. 13 shows the clear distinction between overlaps and their conductivity. Since gold is a highly conductive element, it is expected that a sample containing more of it would have an enhanced conductivity. Previous studies developing a transistor have found that gold nanoparticles resulted in improved electrical performances [15]. The sample sputtered for 8 min with gold resulted in a higher conductivity than that sputtered for only 4 min. Samples with two overlaps are shown to have a lower conductivity than samples with one overlap as shown in Fig. 13. As previously denoted, the gold concentration decreased with increasing overlaps, hence explaining the reduction in conductivity. This is also supported in previous studies using gold sputtering techniques on glass, where the sheet resistance of the latter decreased exponentially with increasing sputtering time [28]. Since air is a poor conductor of electricity, it is expected that the samples with two overlaps would have a lower conductivity due to their increased oxygen concentrations previously determined from the EDX results.

Most of the conduction can be explained through quantum effects due to the dispersion and distance between the gold particles. Assuming the particles are of a spherical shape and the matrix is insulating, then the volume fraction can be determined as in Eq. 6, where *R*_*c*_ is the conductive particle radius, *R*_*i*_ the insulating particle radius, and *n*_*c*_ and *n*_*i*_ are the number of conductive and insulating particles, respectively [29].

6 $$ P=\frac{n_c{R}_c^3}{n_c{R}_c^3+{n}_i{R}_i^3} $$

The previous measurements of particle sizes acquired from the TEM images were used to determine the volume fraction of the conductive phase, *P*. These results can then be used in conjunction with Eq. 7 to determine the theoretical interparticle distances, *l*, assuming spherical conductive particles and a uniform size distribution [30].

7 $$ l={R}_c{\left[\frac{4\pi}{3 P}\right]}^{1/3}-2 $$

The interparticle distance can then be related directly to the conductivity of the silicon oxide *σ*_*i*_ and gold particles *σ*_*c*_ as in Eq. 8 below [31].

8 $$ {\sigma}_i={\sigma}_c{e}^{-2{X}_t l} $$

Where is *X*_*t*_ defined as in Eq. 9, with *m* being the mass of the charge carriers, *V*(*t*) the temperature modified barrier height, and *h* is Planck’s constant.

9 $$ {X}_t={\left[\frac{8{\pi}^2 mV(t)}{h^2}\right]}^{0.5} $$

**Fig. 14:**
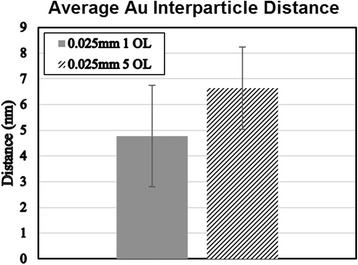
Experimental interparticle distances of gold sputtered silicon samples for 1 OL and 5 OL

Assuming constancy of the parameters in *X*_*t*_, the effect on the conductivity of the silicon oxide becomes highly dependent on the distance between the conductive particles. As one would expect from the equations, higher numbers and larger particle radii of conductive particles results in a higher volume fraction, which in turn results in increases in interparticle distances. From the measured particle sizes depicted earlier in Fig. 11, the relationship between the gold particle radii and the conductivity agree with the theoretically proposed relationships. The greater the distance between the conductive gold particles, the lower the overall conductivity of the silica. As seen in Fig. 14, the gold particle distances increase with a decrease in overlap, further agreeing with the conductivity measurements expected.

## Conclusions

In this report, a method of nanofiber generation using a nanosecond pulsed laser is proposed along with a technique to customize the electrical properties of laser processed silicon to improve its viability in sensing applications requiring a biocompatible environment using gold sputtering techniques. Micro and nanofibrous structures were achieved using a nanosecond Nd:YAG pulsed laser system on a single crystalline silicon wafer. Laser pulses enable to precisely deliver large amounts of energy into the surface of a material in order to achieve a desired nanofibrous structures. For silicon as an opaque material, the laser energy is absorbed near the surface, synthesizing thin-film of nanofibrous silicon without altering the bulk properties. The processed silicon samples were sputtered with gold for duration of either 4 or 8 min to impart and compare its effects on the conductive properties. Overlap number and line spacing were varied in this experiment, and the changes in the absorption capabilities of the samples were experimentally measured and compared. The absorption was found to increase at smaller line spacings and at higher overlaps, allowing for the rearrangement of the silicon substrate into fibers and agglomerates capable of absorbing more light. It was shown that both gold and silicon particles exhibited growth as the absorption coefficients of the materials increased. Fibrous structures were seen to form at shorter line spacings and at higher powers. As the overlap numbers were increased, the fiber diameters grew as well due to the growth in particle sizes. Finally, the conductivity showed some controllability in terms of the duration of sputtering undergone by the samples.

Identifying the fabrication technique for such biocompatible sensor devices is vital and is still being in progress. More studies, in current future direction of this project, need to be conducted to distill the proposed method and propose the guidelines to ascertain the scientific challenges as well as the prerequisites to make this technology market-viable. Although there is yet more research to be done in this area, these findings act as an important preliminary review as to the direction in which biological sensing surfaces can be further adapted and made cost effective. Silicon, being a semiconductor and one of the most common resource for electronic and circuit building, can now impart conductive and biocompatible properties. This method outlines an economic, simple, and yet effective way to process silicon to achieve nanofibrous structures able to increase its biocompatibility while still allowing for electrical conductance.

## References

[1] Shi J, Porterfield DM (2011) Surface modification approaches for electrochemical biosensors. INTECH Open Access Publisher

[2] He P, Dai L (2006) Carbon nanotube biosensors. *BioMEMS and biomedical nanotechnology*, Springer US, p 171-201

[3] Yang N, Chen X, Ren T, Zhang P, Yang D (2015). Carbon nanotube based biosensors. Sensors Actuators B Chem.

[4] Balasubramanian K, Burghard M (2006). Biosensors based on carbon nanotubes. Anal Bioanal Chem.

[5] Eatemadi A, et al (2014) Carbon nanotubes: properties, synthesis, purification, and medical applications. Nanoscale Res Lett 9(1):393. doi:10.1186/1556-276X-9-393. https://nanoscalereslett.springeropen.com/articles/10.1186/1556-276X-9-393 10.1186/1556-276X-9-393 PMC4141964 25170330

[6] Davis JR (2003). Overview of biomaterials and their use in medical devices. *Handbook of materials for medical devices*.

[7] Jamois C, Li C, Gerelli E, Orobtchouk R, Benyattou T, Belarouci A, Chevolot Y, Monnier V, Souteyrand E (2011) New concepts of integrated photonic biosensors based on porous silicon. Biosensors-Emerging Materials and Applications

[8] Dhanekar S, Jain S (2013). Porous silicon biosensor: current status. Biosens Bioelectron.

[9] Buckberry L, Bayliss S (1999). Porous silicon as a biomaterial. Mater. World.

[10] Jones MH, Jones SH (2003). Wet-chemical etching and cleaning of silicon.

[11] Qiu ZY, Chen C, Wang XM, Lee IS (2014). Advances in the surface modification techniques of bone-related implants for last 10 years. Regenerative Biomaterials.

[12] Colpitts C, Ektesabi AM, Wyatt RA, Crawford BD, Kiani A (2016). Mammalian fibroblast cells show strong preference for laser-generated hybrid amorphous silicon-SiO2 textures. J Appl Biomater Funct Mater.

[13] Kiani A, Venkatakrishnan K, Tan B (2009). Micro/nano scale amorphization of silicon by femtosecond laser irradiation. Opt Express.

[14] Mitra SK, Saha AA (2015) Surface modification, methods. *Encyclopedia of Microfluidics and Nanofluidics*, pp 3115-3123. doi:10.1186/1556-276X-9-393. https://link.springer.com/referenceworkentry/10.1007%2F978-0-387-48998-8_1503

[15] Presnova G, Presnov D, Krupenin V, Grigorenko V, Trifonov A, Andreeva I, Ignatenko O, Egorov A, Rubtsova M (2017). Biosensor based on a silicon nanowire field-effect transistor functionalized by gold nanoparticles for the highly sensitive determination of prostate specific antigen. Biosens Bioelectron.

[16] Chang HY, Arshad MM, Fathil MFM, Hashim U (2016) Gold nanoparticles embedded silicon channel biosensor for improved sensitivity. In Mahmood MR, Soga T, Nagaoka S, Mamat MH, Jafar SM (eds) AIP Conference Proceedings (Vol. 1733, No. 1, p. 020074). AIP Publishing. doi:http://dx.doi.org/10.1063/1.4948892

[17] Zhang W, Du Y, Wang ML (2015). On-chip highly sensitive saliva glucose sensing using multilayer films composed of single-walled carbon nanotubes, gold nanoparticles, and glucose oxidase. Sensing Bio-Sensing Res..

[18] Rahmani M, Lukiyanchuk B, Tahmasebi T, Lin Y, Liew TYF, Hong MH (2012). Polarization-controlled spatial localization of near-field energy in planar symmetric coupled oligomers. Applied Physics A.

[19] Colpitts C, Kiani A (2016). Synthesis of bioactive three-dimensional silicon-oxide nanofibrous structures on the silicon substrate for bionic devices’ fabrication. Nanomaterials Nanotechnology.

[20] Tavangar A, Tan B, Venkatakrishnan K (2013). Study of the formation of 3-D titania nanofibrous structure by MHz femtosecond laser in ambient air. J. Appl. Phys..

[21] Gaharwar AK, Sant S, Hancock MJ, Hacking SA (eds) (2013) Nanomaterials in tissue engineering: fabrication and applications. Elsevier. doi:10.1533/9780857097231.1

[22] Kiani A, Patel NB, Tan B, Venkatakrishnan K (2015). Leaf-like nanotips synthesized on femtosecond laser-irradiated dielectric material. J Appl Phys.

[23] Tan B, Venkatakrishnan K (2009). Synthesis of fibrous nanoparticle aggregates by femtosecond laser ablation in air. Opt Express.

[24] Brown MS, Arnold CB (2010). Fundamentals of laser-material interaction and application to multiscale surface modification. In Laser precision microfabrication (pp. 91-120). Springer Berlin Heidelberg. doi:10.1007/978-3-642-10523-4_4

[25] Singh N, Razafinimanana M, Gleizes A (1998). The effect of pressure on a plasma plume: temperature and electron density measurements. J Phys D Appl Phys.

[26] Kiani A, Venkatakrishnan K, Tan B (2010). Direct laser writing of amorphous silicon on Si-substrate induced by high repetition femtosecond pulses. J Appl Phys.

[27] Gamaly EG, Rode AV, Luther-Davies B (1999). Ultrafast ablation with high-pulse-rate lasers. Part I: theoretical considerations. J Appl Phys.

[28] Aroutiounian VM, Ghulinyan MZ (2003). Electrical conductivity mechanisms in porous silicon. Phys Status Solidi A.

[29] Mukherjee R, (2014). Correlation effects in nanoparticle composites: percolation, packing and tunneling

[30] Özmıhçı FÖ, Balköse D (2013). Effects of particle size and electrical resistivity of filler on mechanical, electrical, and thermal properties of linear low density polyethylene–zinc oxide composites. J Appl Polym Sci.

[31] Athanassiou EK, Krumeich F, Grass RN, Stark WJ (2008). Advanced piezoresistance of extended metal-insulator core-shell nanoparticle assemblies. Phys Rev Lett.

